# War surgery in Afghanistan: a model for mass causalities in terror attacks?

**DOI:** 10.1007/s00264-020-04797-2

**Published:** 2020-09-11

**Authors:** F. Wichlas, V. Hofmann, G. Strada, C. Deininger

**Affiliations:** 1 Department of Orthopedics and Traumatology, Paracelsus Medical University, Müllner Hauptstrasse 48, 5020 Salzburg, Austria; 2 Emergency NGO, Milan, Italy

**Keywords:** Low-income country, War surgery, Trauma surgery

## Abstract

**Purpose:**

The aim of the study was to identify solution strategies from a non-governmental (NGO) hospital in a war region for violence-related injuries and to show how high-income countries (HIC) might benefit from this expertise.

**Methods:**

NGO trauma hospital in Lashkar Gah, Afghanistan. Four hundred eighty-four war victims admitted in a three month period (February 2016–May 2016) were included. Patients´ characteristics were analyzed.

**Results:**

The mean age was 23.5 years. Four hundred thirty-four (89.9%) were male, and 50 (10.1%) were female. The most common cause of injury was bullet injuries, shell injuries, and mine injuries. The most common injured body region was the lower extremity, upper extremity, and the chest or the face. Apart from surgical wound care and debridements, which were performed on every wound in the operation theatre, laparotomy was the most common surgical procedure, followed by installation of a chest drainage and amputation.

**Conclusion:**

The surgical expertise and clear pathways outweigh modern infrastructure. In case of a mass casualty incident, fast decision-making with basic diagnostic means in order to take rapid measurements for life-saving therapies could make the difference.

**Electronic supplementary material:**

The online version of this article (10.1007/s00264-020-04797-2) contains supplementary material, which is available to authorized users.

## Introduction

Medical care in low-income countries (LIC) differs a from western medical standards [1]. Compared with civilian trauma in LIC, which is mainly caused by road traffic accidents, the injuries in war zones present different patterns with numerous wounds caused by bullets, mines, and bombs [2, 3].

In high-income countries (HIC), the surgical training focuses early on a specialty. This leads to high knowledge in a very narrow surgical field but a lack of broad general surgical experience [4–6].

The lack of surgical experience might not be relevant as long as a hospital provides a specialist for every probable pathology, but in cases of a sudden high volume of causalities like in a terror attack or train accident, adequate treatment of the injured could get difficult [7, 8]. In this setting, a specialist for every injured region in one patient would deplete human resources.

Besides fast surgery in mass casualties, patients’ flow needs to be efficient, both in speed and direction. The in-hospital pathways must be clear for the personnel from the moment the patient enters the hospital to the final destination [9].

As much as medical standards in LIC and war zones lag behind, there might be a potential knowledge of primary injury treatment and basic surgical techniques, expectable injury patterns, and experience in dealing with mass causalities by fast decision-making. A hospital in a war region has limited resources and needs to cope with a high constant and sudden patient inflow. The surgeons in these hospitals would have an incomparable amount of experience in treating war injuries. The question is, if this knowledge can be helpful when an unexpected incident like a terror attack happens in a “developed HIC” country.

The aim of the study was to identify possible solution strategies from a non-governmental (NGO) hospital in a war region, in Lashkar Gah, Afghanistan. For this purpose, we analyzed the hospital resources, its management strategies, and the epidemiology of war injuries and their treatments. Useful pathways and surgical skills for HIC should be determined to help cope with mass casualties and terror injuries. The hypothesis was that, when a hospital with limited resources could treat a high amount of war injuries, the important factors must lie somewhere else than on the resources.

A solution strategy for HIC might be to develop a guideline for fast decision-making with the simplest diagnostic means in order to treat first what kills first with the quickest and safest treatment option available.

## Material and methods

### Setting

The NGO hospital is equipped with 92 beds, six intensive care unit (ICU) beds without ventilator, two operation theatres (OT), and one outpatient department (OPD). Besides the six wards, there was a room for physiotherapy and casting. The OT have swing doors and are placed near the OPD (short ways). Although the OT is clean, the hygienic level is very basic compared with western standards. Intra-operative X-ray control is difficult but feasible. The hospitals X-ray machine is analogue and no CT is available.

In total, four junior and four trauma senior surgeons were in charge; during on calls one junior and one senior surgeon were present. Besides the surgeons, the medical staff had a questionable education concerning medical health college or even school. They are mainly directly instructed at the hospital and have very specifically defined duties. Anaesthesia is performed by “anesthiologic technicians” trained by international aestheticians, they are not anaesthetists.

The international team was a general surgeon, an orthopaedic/trauma surgeon (the author), and an anesthesiologist. Logistics and teaching nurses were provided by the NGO.

Among admitted patients were war victims, civilian trauma victims younger than 14 years old, and patients in life-threatening condition of any sort. Patients with chronic post-traumatic deformities were not admitted [10].

### Management and hospital pathways

The triage and management of injured patients were done by OPD nurses. The standardized procedure consisted of the measurement of heart rate, blood pressure, oxygen saturation, and the clinical examination after undressing and cleaning the patient. Two intravenous lines were inserted, and blood samples for laboratory analysis were taken. Fluid resuscitation was usually done with 2 l Ringer’s lactate. Packed red blood cells were available.

The national surgeon completed diagnostics by clinical examination, auscultation of chest and abdomen, and seldom ordering an X-ray. Subsequently the patients went for operation without any delay. In the OT, the patient was draped and intubated for surgery by the local staff before the surgeon could even scrub entirely. The patients flow was clearly defined from OPD to OT and then to the ward or ICU for minor and life-threatening injuries. It was performed very fast by the local staff for both.

### War surgery

Surgery and treatment of war wounded was performed according war surgery guidelines 10 [11].

### Epidemiology

Data for evaluation was collected from 26.02.2016 to 11.05.2016. In this 76 days period, 577 patients were admitted to the hospital, 484 (83.88%) war victims, 77 (13.35%) children that had sustained falls or road traffic accidents, and 16 (2.77%) patients of miscellaneous, life-threatening injuries. Patients mean age was 21.84 years, most of them were male (Table 1).

**Table 1:** Characteristics of all admitted patients during the study period

	*n* (%)	Mean age in years (SD)	Gender	
			Male (%)	Female (%)
All	577	21.84 (13.15)	512 (88.73)	65 (11.27)
War victims	484 (83.88)	23.48 (12.58)	434 (89.67)	50 (10.33)
Falls/road traffic accidents	77 (13.34)	9.39 (8.86)	64 (83.12)	13 (16.88)
Miscellaneous	16 (2.77)	8.63 (9.10)	14 (87.50)	2 (12.50)

For this study, only war victims were evaluated (*n* = 484). The mean age of war casualties was 23.5 years; 434 (89.9%) were male, and 50 (10.1%) were female.

The injuries were analyzed for the cause of injury, the region injured, for the surgical procedures performed, and the death rate.

Of all patients, 233 were readmitted for planned surgery, and four were readmitted twice. Planned suregry was mostly delayed primary closures.

## Results

### Cause of injury and affected body region of war victims

All regions of the body were affected: skull, face, eye, neck, chest, abdomen, back, flank, buttock, pelvis, genitourinary, spine, upper extremities (UE), and lower extremities (LE).

The most common cause of injuries were bullet injuries (BI) *n* = 282 (58.3%) followed by shell injuries (SI) *n* = 137 (28.3%), mine injuries (MI) *n* = 44 (9.1%), and stab wounds (SW) *n* = 21 (4.3%).

The most common injured body region was the LE followed by the UE and the chest or the face.

**Fig. 1:**
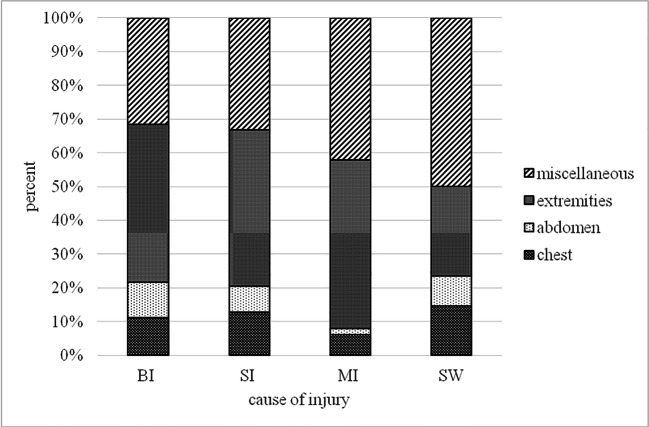
Cause of injury and affected body region in percent. 282 BI (bullet injury), 137 SI (shell injury), 44 MI (mine injury), and 21 SW (stab wound)

An overview dealing with the cause and the affected body region can be seen in Fig. 1 and Table 2.

**Table 2:** Cause of injury and affected body region in percent. BI (bullet injury), SI (shell injury), MI (mine injury), SW (stab wound), LE (lower extremities), and UE (upper extremities)

	Injured body region														
Cause of injury	Skull	Face	Eye	Neck	Chest	Abdomen	Pelvis	Back	Spine	Flank	Buttock	Genitourinary	UE	LE	All
BI	4.85%	4,08%	0.77%	1.53%	11,22%	10.46%	4.08%	2,55%	1.28%	4.85%	6,38%	1.28%	20.92%	25,77%	100.00%
SI	5.63%	7.62%	4.30%	3.97%	12.91%	7.62%	1.66%	3.97%	0.33%	1.99%	3.31%	0.33%	21.52%	24.83%	100.00%
MI	7.14%	12.50%	5.36%	3.57%	6.25%	1.79%	0.00%	1.79%	0.89%	1.79%	0.89%	8.04%	18.75%	31.25%	99.99%
SW	26.47%	11.76%	2.94%	2.94%	14.71%	8.82%	0.00%	2.94%	0.00%	2.94%	0.00%	0.00%	20.59%	5.88%	100.00%

### Performed procedures

A surgical wound debridement was performed on every patient that got operated upon. All war wounds were left open at the primary operation and planned for delayed primary closure five days later [11]. No wound dressing was made before that fifth day in the operation room, except there was a high suspicion for infection. If the health condition allowed, the patient was discharged and readmitted for delayed primary closure. In case of clean stab wounds, they were closed primarily after debridement.

The second most common specific surgical procedure performed was the laparotomy *n* = 66 (30.70%) (additional 4 revision operations) followed by installation of a chest drainage *n* = 40 (18.60%) and amputation *n* = 29 (13.48%). Two hundred fifteen war injuries underwent further surgical procedures. In average 2.83 operations were performed and 6.6 war injured were admitted per day. Taking into account the fact that all wounds have been debrided in the operation theatre, the average number of surgical procedures increases to 6.37 per day.

Broken down into injured body regions: After BI the most common operation was the laparotomy *n* = 40 (34.48%) followed by installation of a chest drainage *n* = 25 (21.55%) and vascular reconstruction/craniotomy *n* = 9 (7.76%). After SI the most common operation was the laparotomy *n* = 20 (35.71%) followed by installation of a chest drainage *n* = 9 (16.07%) and vascular reconstruction *n* = 6 (10.71%). After MI the most common operation was the amputation *n* = 19 (51.35%) followed by installation of a chest drainage *n* = 5 (13.51%) and laparotomy *n* = 3 (8.11%). After SW the most common operation was the laparotomy *n* = 3 (50.00%) followed by chest drainage/amputation/vascular reconstruction *n* = 1 (16.67%).

**Fig. 2:**
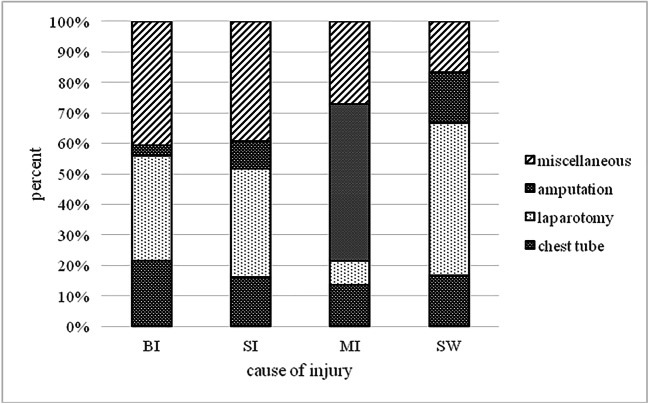
Cause of injury and surgical procedure performed in percent. BI (bullet injury), SI (shell injury), MI (mine injury), SW (stab wound), UE (upper extremities), LE (lower extremities), LAP (laparotomy), TU (chest tube placement), AMP (amputation), and miscellaneous procedures are shown in Table 3

An overview over the cause of injury and the surgical procedures performed can be seen in Fig. 2 and Table 3.

**Table 3:** Cause of injury and surgical procedure performed in percent. BI (bullet injury), SI (shell injury), MI (mine injury), SW (stab wound), UE (upper extremities), LE (lower extremities), LAP (laparotomy), TU (chest tube placement), AMP (amputation), VR (vessel repair, CRAN (craniotomy), VL (vessel ligation), TCT (thoracotomy), TRACH (tracheotomy), GAST (gastrostomy), TRACT (application of a traction device), K-WIRE (reduction and K-wire fixation), and EX FIX (reduction and external fixation)

	LAP	TU	AMP	VR	CRAN	VL	TCT	TRACH	GASTR	TRACT	K-WIRE	EX FIX	All
				Miscellaneous									
BI	34.48%	21.55%	3.45%	7.76%	7.76%	6.90%	6.03%	0.86%	0.86%	6.03%	2.59%	1.72%	100.00%
SI	35.71%	16.07%	8.93%	10.71%	7.14%	1.79%	0.00%	5.36%	3.57%	3.57%	3.57%	3.57%	100.00%
MI	8.11%	13.51%	51.35%	0.00%	2.70%	2.70%	0.00%	5.41%	2.70%	5.41%	5.41%	2.70%	100.00%
SW	50.00%	16.67%	16.67%	16.67%	0.00%	0.00%	0.00%	0.00%	0.00%	0.00%	0.00%	0.00%	100.00%

In 60 cases (12.40%), the injuries were located on both sides of the body. The percentage was maximal for MI (50.00%), followed by SI (18.98%) and BI (4.26%).

### Laparotomies

Analyzing the 66 primary laparotomies, the most often injured intra-abdominal organ found was the bowel (47.41%) followed by the liver (11.20%) and the diaphragm (8.62%). A laparotomy was performed on all perforating abdominal injuries. Seven of these diagnostic laparotomies were negative.

In BI and SI, the bowel was injured the most, followed by the diaphragm. Third most common injured organs were the liver and kidney for BI and liver and spleen for SI. Concerning the abdomen, mines injured the bowel only.

Accordingly, the most frequently performed procedures were bowel-related (direct repairs 44.33%, anastomosis 14.67%, loop colostomies 8.00%, and colectomies 8.00%). They were followed by emergency procedures (5.33% packing, 5.33% splenectomies and 5.33% aortic clamping). For BI the second most common procedures were splenectomies, nephrectomies, and aortic clamping and for SI, packing, splenectomies, and aortic clamping.

### Bony procedures

Bones were fractured in 166 patients of 484 (34.30%), 95 BI patients of 282 (33.69%), 39 SI patients of 137 (28.46%), 31 MI patients of 44 (70.45%), and one SW of 21 (4.76%). Most fractures were recorded in MI patients, followed by BI and SI. In total we treated seven patients with K-wire, five with fixateur externe, and eleven with bone traction. All other fractures were treated conservatively. One example of a conservatively treated femoral shaft fracture due to a BI is shown in images 1 and 2.

Amputations accounted for 43.18% of 44 MI patients (*n* = 19), 3.65% of 137 SI patients (*n* = 5), 1.42% of 282 BI patients (*n* = 4), and 4.76% of 21 SW patients (n = 1). Most amputations were caused by MI, followed by SI and BI.

We readmitted 216 (44.63%) patients, 50.71% of BI, 43.07% of SI, 27.27% of MI, and 9.52% of SW. Of these readmitted patients, 56.96% had an injury of the UE or LE. Most of the injuries were readmitted for delayed primary closure (81.02%) and discharged the same day (71.30%).

### Death rates

In total, *n* = 14 (2.89%) patients died in the hospital; these were 11 out of 282 BI patients (3.90%), one out of 138 SI patients (0.73%), one out of 44 MI patients (2.72%), and one out of 21 SW patients (4.76%).

## Discussion

Trauma surgery in a war zone such as Lashkar Gah differs from trauma surgery in a HIC. Thus, there is a lot of knowledge to benefit from, especially when dealing with uncommon injuries and mass causalities [12].

### Pathway and education

Although the Lashkar Gah hospital has very limited equipment, a high patient inflow can be managed. This NGO hospital cannot be compared with a military facility, as both the financial means and the training of the medical staff working there are completely different. In addition, it is extremely difficult to transfer severely injured patients to a larger trauma centre. Nevertheless, with these resources on one day (21.03.2016), 22 Patients (21 BI) were treated by four surgeons within 24 h without enabling mass casualty protocol. In this 92-beds-hospital, this amount equals 23.91% of the overall capacity. This becomes even more impressive, regarding the fact that with exception of the surgeons, the medical personnel was trained in the hospital only, with no pre-existing medical education. It seems that standardized pathways can compensate the lack of medical education and are crucial in the treatment of high patient’s inflow.

According to Lesaffre et al., who analyzed the terror attacks in Paris 2015, a “…simpler and more robust organization…” is one of the most significant factors to deal with a mass casualty incident [9]. During the Paris Terror in 2015, 495 wounded and 130 dead victims were counted. One hundred twenty-four before reaching a hospital. This day, about 1800 firefighters trained in first aid were on call. Many of the 7900 Parisian firefighters are accommodated in 80 fire stations and thus quickly accessible. In case of a terror attack, the severely injured patient may not reach a level 1 trauma centre, but a primary health care institution. This was the case in 22% of the absolute emergencies in Paris 2015. This fact underlines the need of a profound training in life-saving skills of every doctor in charge [9]. In those cases, necessary diagnostic procedures should be kept simple and focused on life-threatening injuries to assure a quick life-saving treatment. They include a fast but thorough clinical examination of the undressed patient, measurement of heart rate, blood pressure, and oxygen saturation, insertion of two large bore intravenous lines, taking blood samples, and radiography only if necessary. All these steps stick to a well-defined simple pathway.

After diagnosing, a fast treatment without delay is essential. Besides fast and thorough wound debridement, this means mostly performing laparotomies, inserting chest tubes and amputations. These skills are crucial to save the patient’s life [9, 13]. They are also providing the basis for western surgeons dealing with an enormous number of casualties by a terror attack or a mass catastrophe [14, 15]. The speed factor is essential, not only for the patient treated momentarily, but for the next severely injured patients waiting for treatment.

Surgical skills needed include vessel repairs, craniotomies, and thoracotomies.

The war surgeon needs to combine techniques from different surgical fields such as maxilla-facial, plastic, abdominal, orthopedic, and neurosurgery [4, 16]. In Lashkar Gah, specialist surgical care was needed for eye related procedures. These patients were sent to Kabul.

Although war surgery’s spectrum is broad, surgeons have to deal with two main trauma mechanisms. Most of the injuries are caused by penetrating high velocity projectiles of any sort (BI and SI) or by blasts and burns from explosives like bombs or mines (SI and MI) [2].

Even though resources in western countries might be superior to NGO hospitals in LIC, critical incidents like terror attacks will overwhelm local resources [17]. As a solution, mass casualty protocols have been created to assure a proper medical health care even in situations with more than 500 severely injured [9, 18].

### Skills

Besides these in-hospital pathways, surgical skills need to be trained as well. Teaching of life-saving procedures, compulsory for every surgeon in training might be helpful [19, 20], but they hardly match the surgeons expertise gained through high patient turnover. But not every patient is “in extremis.” Wound debridements, simple laparotomies, insertion of chest tubes, and vessel repairs are probably the majority of cases and need to be achieved as fast as possible.

Treatment pathways and surgical skills are the main characteristics of the Lashkar Gah hospital for civilian war victims. Although medical education was not available for most of the personnel and the hospital is very basic, it could cope with a high number of patients. Training, surgical skills, and clear pathways did lead to reliable, appropriate, and rapid treatment of the seriously injured, even with a large proportion of staff who have not received any official medical training.

### Limitations

The mission period was restricted to three months, and the data collected only reflect this period. Due to the limited documentation in a war zone, a further evaluation of long-term follow-up was not possible. However, the necessity to prepare for an extremely seldom incident, such as a terror attack, can be discussed. Although medial impact is increasing, death due to terrorism has decreased over the last 40 years [21]. Further, the data published from these incidents showed good coping strategies of the treating hospitals, even without specific preparation [22]. Nevertheless, the determined characteristics could be useful for polytraumatized patients. In these cases, fast treating algorithms and surgical manoeuvres are essential [9].

Summarizing, the knowledge of any anatomic region and the ability to perform fast surgery make the war surgeon unique. Speed is essential in surgery and treatment pathways. Those special abilities can provide a basis for surgeons working in HIC who are confronted with a mass casualty incident like a terror attack.

The lesson learned from Lashkar Gah for terror surgery in Europe:

- Surgeons must be trained in war surgery performing thorough debridements, laparotomies, chest tube insertions, vessel repair, and craniotomies.
- Treatment pathways must be trained by the hospitals’ staff.
- The hospitals´ resources are of minor importance.

## Electronic supplementary material

ESM 1 (DOC 5011 kb)

ESM 2 (DOC 4185 kb)
