# Supplementary material for: War surgery in Afghanistan: a model for mass causalities in terror attacks?
Source: Int Orthop. 2020 Sep 11;44(12):2521–7. doi: 10.1007/s00264-020-04797-2 (PMC7483489; doi:10.1007/s00264-020-04797-2)

**Image 2**- X-Ray in two planes of a femoral shaft fracture due to a BI of a 26 years old male patient after conservative treatment with Perkins traction after debridement and 5 days of casting


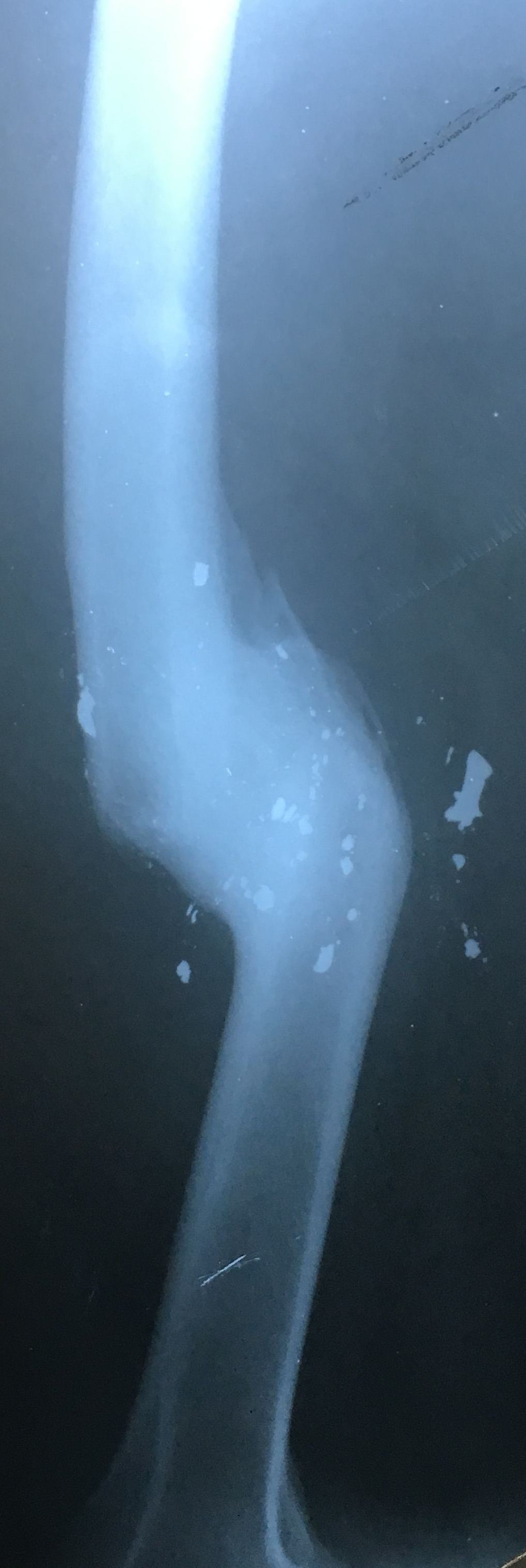

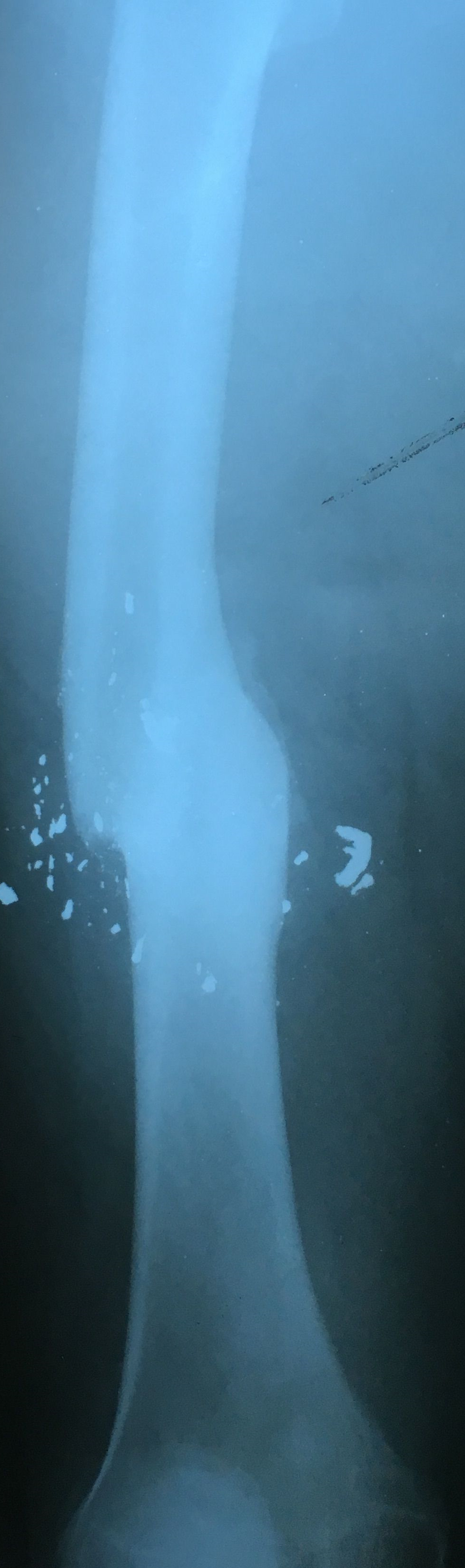

Supplement: Supplementary file 2 — (DOC 4185 kb) [file 264_2020_4797_MOESM2_ESM.doc]
